# Meeting report: the 5th International expert symposium in Fukushima on radiation and health

**DOI:** 10.1186/s12940-017-0211-y

**Published:** 2017-01-18

**Authors:** Vladimir A. Saenko, Geraldine A. Thomas, Shunichi Yamashita

**Affiliations:** 1 Department of Radiation Molecular Epidemiology, Atomic Bomb Disease Institute, Nagasaki University, 1-12-4 Sakamoto, 852-8523 Nagasaki, Japan; 2 Imperial College London, Charing Cross Hospital, Fulham Palace Road, London, W6 8RF UK; 3 Radiation Medical Science Center for the Fukushima Health Management Survey, Fukushima Medical University, 1 Hikarigaoka, 960-1295 Fukushima, Japan

**Keywords:** Fukushima, Chernobyl, Radiation, Thyroid cancer, Ultrasound screening

## Abstract

**Background:**

The symposium entitled “Chernobyl +30, Fukushima +5: Lessons and Solutions for Fukushima’s Thyroid Question” was held in September, 2016 in Fukushima. The aim of the Symposium was to revisit and recapitulate evidence from the studies in Chernobyl in order to share multidisciplinary opinions and views on the likely reason for the high rate of thyroid cancer detected by the Thyroid Ultrasound Examination program in Fukushima Prefecture.

**Participants and matters discussed:**

The high prevalence of thyroid cancer in young individuals causes concerns among Fukushima residents and the general public that it might be due to putative radiation exposure from the Fukushima Daiichi Nuclear Power Plant accident. Twenty-six experts from Japan and abroad, including participants affiliated with international organizations, reviewed the results of radiation epidemiology investigations in Chernobyl, presented clinical experience of diagnosis, treatment and follow-up of patients with radiation-related thyroid cancer, and scrutinized the findings on thyroid cancer in Fukushima.

**Conclusion:**

Conclusions drawn at the symposium included understanding that in contrast to Chernobyl, doses to the public from the accident in Fukushima were too low to give rise to a discernible excess risk for thyroid cancer. The high detection rate of thyroid cancer and benign abnormalities resulted from the use of highly sensitive ultrasound equipment and sophisticated protocol of examination used in the Thyroid Ultrasound Examination, and therefore not attributable to radiation. Coordinated efforts will be necessary to avoid overdiagnosis and overtreatment, which may carry its own health disbenefits. Clear communication to the screening participants and their families is recommended in regard to why the examination is being conducted and to explain the likely outcomes and risks, including the means and options for treatment if a thyroid disorder is detected.

**Electronic supplementary material:**

The online version of this article (doi:10.1186/s12940-017-0211-y) contains supplementary material, which is available to authorized users.

## Background

Two thousand sixteen marks the 30th anniversary of the Chernobyl and the 5th anniversary of the Fukushima Daiichi Nuclear Power Plant (NPP) accidents. These two dramatic events in different countries, separated in time by 25 years, have been rated 7th level, i.e., a “Major accident”, according to the International Atomic Energy Agency’s (IAEA) International Nuclear and Radiological Event Scale [1]. Although atmospheric releases of radionuclides have been eventually determined to be at least an order of magnitude lower in Fukushima than those in Chernobyl [2], an uncertainty in potential exposure doses during the acute phase of the crisis in Fukushima led to emergency response, which initially involved evacuation of some 165,000 residents living in the 30 km zone around the plant. The potential of an involuntary exposure to radiation, and concerns about its potential health effects, resulted in the establishment of the Fukushima Health Management Survey Program [3], one of four components of which is the Thyroid Ultrasound Examination (TUE) launched in October 2011 [4]. The high prevalence of thyroid cancer among young individuals aged 0–18 years as of April 1, 2011 in TUE [5] detected by the sensitive methods further augmented fears and anxiety about the relationship of thyroid cancer to radiation in Fukushima. Since the studies on health consequences of the Chernobyl accident in exposed population performed during the last 30 years are a unique source of knowledge to assess whether an evidence-based link between radiation releases and thyroid cancer in young residents in Fukushima may exist, the Symposium convened on September 26–26, 2016, revisiting data from Chernobyl and Fukushima from the point of view of different scientific disciplines to offer insights into the reasons for the seemingly high detection rate of thyroid cancer in Fukushima. Lists of the Organizing Committee members, and of the chairpersons and participants of the Symposium are provided in Additional file 1 and Additional file 2, respectively.

## Thyroid cancer in the regions affected by the Chernobyl accident

The Chernobyl studies reviewed included those on epidemiology and long-term clinical outcomes of treatment of young patients with thyroid cancer.

Detectable excess risk for thyroid cancer as a result of radiation exposure in the individuals aged less than 18 years at the time of the Chernobyl accident is well-established to exist. It is for this reason that the age of 18 at the time of the accident was used to determine the age inclusion criterion in Fukushima TUE. Prospective cohort studies in Ukraine, Belarus and Russia concur that the risk remains elevated even until today, i.e., 30 years after the accident. However, as the UkrAm study shows, excess relative risk per dose unit is declining with time in each consequent round of ultrasound screening [6], and is accompanied by time-dependent pathomorphological evolution of tumor characteristics to the less aggressive phenotype with corresponding molecular genetic changes [7]. In the individuals exposed at young age in Ukraine and Russia who then developed thyroid cancer, mean radiation doses to the thyroid ranged 0.4–3.4 Gy or exceeded 0.2 Gy, respectively [6, 8], which is an important fact in view of low thyroid doses in Fukushima [9–11]. Also of relevance to the Fukushima scenario, a significant screening effect was found in the Chernobyl cohort, accounting for a 6.74-fold increase, on average, in the detection rate of thyroid cancer during the 20-year long period of observation since 1991. The size of screening effect appears to decrease with time being the highest during the first 5 years of the study. If it was postulated that a similar effect on thyroid cancer rate would be observed in Fukushima, it could be predicted that the frequency of expected thyroid cancers occurring as a result of the TUE would be similar to those actually observed [12].

Pathological studies indicate that radiation-induced thyroid cancers may display more aggressive morphological features than sporadic tumors in age-matched groups of patients, especially among children [13]. The major differences between radiogenic and sporadic papillary thyroid carcinoma, which is the only type of thyroid cancer whose incidence increased sharply after the Chernobyl accident in young patients, include the lower female-to-male ratio and a higher rate of distant metastases in radiogenic cancer, and a higher frequency of co-existing thyroid diseases in sporadic tumors [13, 14]. Importantly, however, the higher morphological aggressiveness of radiation-induced thyroid cancer does not bring about a worse clinical outcome. Indeed, radiation history does not appear to significantly affect long-term treatment results, provided an appropriate, not principally different from that for sporadic thyroid cancer treatment and follow-up had been performed [15]. Despite the advanced stage of disease and sub-optimal initial management, response to radioiodine therapy and final outcomes are favorable in the vast majority of Chernobyl patients [16]. Results of long-term follow-up confirm overall good prognosis and a low frequency of side effects in young individuals receiving high doses of radioiodine therapy for thyroid cancer. Thyroid ultrasound screening may also lead to the earlier detection of smaller tumors and result in a better recurrence-free survival in children and adolescents with PTC.

## Thyroid cancer detected during TUE in Fukushima

Clinical approaches to treating thyroid cancer in Japan and Western countries have some differences. In Japan, a surgical procedure for low-risk thyroid cancer is rather conservative, generally preserving patients’ quality of life, and postoperative management does not widely involve radioiodine therapy. Such a risk-adapted management strategy is based on the analysis of long-term outcomes in patients who had received treatment for thyroid cancer in the country.

The initial screening (1st survey) of the TUE was finished in March 2014, and the full-scale thyroid screening program (2nd survey) – in March 2016; currently the 3rd survey is ongoing since April 2016. The number of patients with malignant or suspected malignant nodules accounted for 116 (among 300,476 examined) and 59 (among 270,327) individuals in the 1st and 2nd surveys, respectively [17, 18].

By the end of September 2016, 127 patients were given thyroid surgery in Fukushima Medical University Hospital. Strict criteria for fine-needle aspiration biopsy are applied to avoid overdiagnosis. For low-risk nodules, including small preclinical thyroid cancers, when regional or distant metastasis is not detected nor extrathyroidal extension is observed, the watch tactics may be recommended. Among the patients with detected thyroid nodules, 39 are now waiting for surgical treatment or preferred nonsurgical observation. A particularity of patients in Fukushima is that they are young, age range 9 – 23 years so far and asymptomatic. Whether the watch-and wait tactics in such young patients, especially in children, is appropriate remains to be determined. There is a real need in preparing guidelines for management of thyroid cancer in children and adolescents in Japan.

Comparison of thyroid cancer cases in Fukushima with data from Chernobyl areas demonstrates a large difference in age distributions of patients from Fukushima and Chernobyl after a period of latency, where the highest risk for thyroid cancer was observed in the individuals of preschool age at exposure [19, 20]. In addition, thyroid doses in Fukushima are also low compared to those in Chernobyl [9–11] and there is no difference in the prevalence of thyroid cancer in different areas of Fukushima Prefecture with different levels of radioactive contamination [5], which was clearly seen in Chernobyl [21]. These circumstances strongly suggest that radiation etiology of these tumors is very unlikely, which is important for the residents of Fukushima Prefecture.

There is a consensus among the professionals in Japan that detecting subclinical thyroid cancer in mass screening of the general population is not beneficial. In part due to this, the increase in thyroid cancer incidence in the country was not as dramatic as in Korea or in the USA. Mass screening inevitably increases the detection rate of thyroid cancer, and it is essential to properly communicate to the population under screening and to consider previous experience of screenings in Japan and other countries [22] to judge about health benefits of currently ongoing programs. Another important point is that TUE was started in response to the demands of Fukushima residents concerned about potential radiation effects on their health. While the specialists and professionals realize that due to biological particularities of well-differentiated thyroid cancer, early diagnosis and treatment of small tumors will unlikely decrease cause-specific mortality, it is essential to deliver this message to the residents in order they better understand benefits and harms of thyroid screening. The current situation reveals a number of challenges, which to be solved need engagement from all stakeholders, advocating of the voluntary basis of participation in health survey, reduction of overdiagnosis, and communication regarding possible disbenefits of overdiagnosis and overtreatment.

## Role of the international organizations

International organizations, including the World Health Organization (WHO), United Nations Scientific Committee on the Effects of Atomic Radiation (UNSCEAR), International Commission on Radiological Protection (ICRP), IAEA and International Agency for Research on Cancer (IARC), have been taking an active part in research post Fukushima since the very beginning of the crisis, and have provided their expertise in evaluation of radiological risks and health effects of the Daiichi NPP accident. The WHO, UNSCEAR and IAEA performed health risk assessment for the population and NPP workers based on the information available at the time of publication [2, 23, 24]. The IAEA report was chronologically the latest and based on the most updated information. Despite some discrepancy in dose estimates between the reports and, consequently, health risk projections, their conclusions are similar: in contrast to Chernobyl, public doses from the accident were low, and occupational doses were within the regulatory limits (an effective dose of 20 mSv per year averaged over five consecutive years, i.e., 100 mSv in 5 years, and of 50 mSv in any single year [25]). Therefore, no immediate health effect attributable to radiation occurred, and no discernible radiation-related health effects are expected in exposed population and their descendants [2, 24].

ICRP has played a prominent role in establishing tight links with Fukushima Medical University to facilitate joint activities on meeting day-to-day challenges that the residents of Fukushima Prefecture face. A key point of ICRP activities has been engaging wide circles of stakeholders in the discussion and decision making process in contaminated areas, emphasizing the role of people exposed to radiation, and bringing in the ethical dimension to the concept of radiological protection.

IARC underscored the role of scientific research in understanding the mechanisms of radiation-related carcinogenesis. Further studies are necessary to identify host and environmental factors that may confound or modify radiation effects on human thyroids, to understand what causes progression or regression of thyroid tumors and to seek molecular signatures of radiation-induced thyroid cancer. All this will contribute to the development of risk-adapted approaches to diagnosis and treatment.

The continuous support of the international organization since the accident has been essential and will be indispensable for the future of recovery in Fukushima Prefecture at all levels in society.

## Conclusions

The Symposium arrived at the following conclusions.

1. The Fukushima Health Management Survey Program is based on surveys with the purpose of long-term health care of prefectural residents and is being carried out in response to their concerns. The health survey was designed to be a study of the health situation of the residents of Fukushima prefecture but it was not intended to be a scientific and epidemiological study of a radiation risk in respect of thyroid cancer. Such an “epidemiological” study would have been long and difficult, would not have responded to the needs expressed by the population and therefore was not considered necessary.
2. Experience from Chernobyl suggests that any increase in thyroid cancer due to radiation exposure would be observed first in those who were very young (0–4 years of age) at the time of the accident, and that any increase would not be observed until 4–5 years after exposure to radiation. It would be predicted therefore that if radiation were the cause of any increase in thyroid cancer in Fukushima, more cases would be observed in the youngest participants of the TUE. To date, thyroid cancers were found in children in their late teenage years, but no cases were found in the most vulnerable group of very young children, which further suggests that the increase is due to a screening rather than radiation exposure. Any cancer takes a period of time from the genotoxic insult that initiates it to detection, even by sensitive techniques such as those used in the TUE. If the increase in thyroid cancer abnormalities seen in the first round of the TUE were to result from exposure to radiation from the accident, an abnormally shorter latency than that observed post Chernobyl would be taking place. This would seem to be a highly unlikely scenario. The proportion of suspicious or malignant cases was almost the same among regions in Fukushima Prefecture. If due to radiation, there should be a relative increase in those areas of the Prefecture with higher radiation exposure.
3. The TUE uses highly sensitive ultrasound sonography equipment for screening the thyroid and routinely detects asymptomatic thyroid abnormalities such as nodules and cysts. Ultrasound thyroid screening of asymptomatic individuals has the potential to do more harm than good to the population, and should only be carried out when clear benefits to the population can be defined; participation in the health surveys and the thyroid screening program must therefore be voluntary. The reasons for the high background rate of thyroid abnormalities, whether malignant or benign, detected in the course of TUE in Fukushima Prefecture in young individuals requires explanation to the non-specialist audience. Clear communication to the screening participants and their families is needed in regard to why the examination is being conducted and the likely outcomes and risks, including the means and options for treatment if a thyroid disorder is detected.

The major message of the Symposium was that the current findings of the TUE in Fukushima Prefecture are not attributable to radiation.

## Additional files

Additional file 1: Members of the Organizing Committee of the 5^th^ International expert symposium in Fukushima on radiation and health (in alphabetical order). (DOC 30 kb)
Additional file 2: Chairpersons and speakers of the 5^th^ International expert symposium in Fukushima on radiation and health (in alphabetical order). (DOC 42 kb)

## Abbreviations

Gy: Gray
IAEA: International Atomic Energy Agency
IARC: International Agency for Research on Cancer
ICRP: International Commission on Radiological Protection
NPP: Nuclear power plant
TUE: Thyroid Ultrasound Examination
UNSCEAR: United Nations Scientific Committee on the Effects of Atomic Radiation
WHO: World Health Organization
