# Supplementary material for: Meeting report: the 5th International expert symposium in Fukushima on radiation and health
Source: Environ Health. 2017 Jan 18;16:3. doi: 10.1186/s12940-017-0211-y (PMC5241991; doi:10.1186/s12940-017-0211-y)
Supplement: Additional file 1: — Members of the Organizing Committee of the 5th International expert symposium in Fukushima on radiation and health (in alphabetical order). (DOC 30 kb) [file 12940_2017_211_MOESM1_ESM.doc]

**Additional file 1**. Members of the Organizing Committee of the 5^th^ International expert symposium in Fukushima on radiation and health (in alphabetical order)

| **Name** | **Affiliation** | **Country** |
| --- | --- | --- |
| Sasakawa Yohei (Chair) | The Nippon Foundation | Japan |
| Gonzalez Abel | Argentine Nuclear Regulatory Commission | Argentine |
| Kikuchi Shin-ichi | Fukushima Medical University | Japan |
| Kita Etsuko | Sasakawa Memorial Health Foundation | Japan |
| Lochard Jacques | International Commission on Radiological Protection |  |
| Mettler Fred | University of new Mexico School of Medicine | USA |
| Ohto Hitoshi | Fukushima Medical University | Japan |
| Yamashita Shunichi | Nagasaki University and Fukushima Medical University | Japan |
