# Supplementary material for: Meeting report: the 5th International expert symposium in Fukushima on radiation and health
Source: Environ Health. 2017 Jan 18;16:3. doi: 10.1186/s12940-017-0211-y (PMC5241991; doi:10.1186/s12940-017-0211-y)
Supplement: Additional file 2: — Chairpersons and speakers of the 5th International expert symposium in Fukushima on radiation and health (in alphabetical order). (DOC 42 kb) [file 12940_2017_211_MOESM2_ESM.doc]

**Additional file 2**. Chairpersons and speakers of the 5^th^ International expert symposium in Fukushima on radiation and health (in alphabetical order)

| **Name** | **Affiliation** | **Country** |
| --- | --- | --- |
| Ahn Hyeong Sik | Korea University School of Medicine | Korea |
| Bogdanova Tetiana | V.P.Komissarenko Institute of Endocrinology and Metabolism | Ukraine |
| Boice John | National Council on Radiation Protection and Measurements | USA |
| Carr Zhanat | World Health Organization |  |
| Clement Christopher | International Commission on Radiological Protection |  |
| Crick Malcolm | United Nations Scientific Committee on the Effects of Atomic Radiation |  |
| Demidchik Yuri | Belarussian Medical Academy of Post-Graduate Education | Belarus |
| Drozd Valentina | Belarussian Medical Academy of Post-Graduate Education | Belarus |
| Gonzalez Abel | Argentine Nuclear Regulatory Commission | Argentine |
| Ivanov Viktor | A.Tsyb National Medical Research Radiation Centre | Russia |
| Kisminiene Ausrele | International Agency for Research on Cancer |  |
| Lochard Jacques | International Commission on Radiological Protection |  |
| Midorikawa Sanae | Fukushima Medical University | Japan |
| Nagataki Shigenobu * | Nagasaki University | Japan |
| Niwa Ohtsura | Radiation Effects Research Foundation | Japan |
| Ohtsuru Akira | Fukushima Medical University | Japan |
| Reiners Christoph | Wurzburg University | Germany |
| Rumiantsev Pavel | Endocrine Research Centre | Russia |
| Saenko Vladimir | Nagasaki University | Japan |
| Sugitani Iwao | Nippon Medical School | Japan |
| Suzuki Shinichi | Fukushima Medical University | Japan |
| Tanigawa Koichi | Fukushima Medical University | Japan |
| Thomas Geraldine | Imperial College London | UK |
| Tronko Mykola | V.P.Komissarenko Institute of Endocrinology and Metabolism | Ukraine |
| Weiss Wolfgang | Federal Office for Radiation Protection | Germany |
| Yamashita Shunichi | Nagasaki University and Fukushima Medical University | Japan |

* to our deep regret, passed away on November 12, 2016, at the age of 84
